# Biomolecular Interaction Studies Between Cytochrome PpcA From *Geobacter sulfurreducens* and the Electron Acceptor Ferric Nitrilotriacetate (Fe-NTA)

**DOI:** 10.3389/fmicb.2018.02741

**Published:** 2018-11-16

**Authors:** Marisa R. Ferreira, Carlos A. Salgueiro

**Affiliations:** UCIBIO-REQUIMTE, Departamento de Química, Faculdade de Ciências e Tecnologia, Universidade NOVA de Lisboa, Caparica, Portugal

**Keywords:** biomolecular interactions, electron transfer, iron respiration, *Geobacter*, multiheme cytochrome

## Abstract

*Geobacter sulfurreducens* bacterium exhibits an enormous respiratory versatility, including the utilization of several toxic and radioactive metals as electron acceptors. This versatility is also replicated in the capability of the most abundant cytochrome in *G. sulfurreducens*, the periplasmic triheme cytochrome PpcA, to reduce uranium, chromium and other metal ions. From all possible electron transfer pathways in *G. sulfurreducens*, those involved in the iron reduction are the best characterized to date. Previously, we provided structural evidence for the complex interface established between PpcA and the electron acceptor Fe(III)-citrate. However, genetic studies suggested that this acceptor is mainly reduced by outer membrane cytochomes. In the present work, we used UV-visible measurements to demonstrate that PpcA is able to directly reduce the electron acceptor ferric nitrilotriacetate (Fe-NTA), a more outer membrane permeable iron chelated form. In addition, the molecular interactions between PpcA and Fe-NTA were probed by Nuclear Magnetic Resonance (NMR) spectroscopy. The NMR spectra obtained for PpcA samples in the absence and presence of Fe-NTA showed that the interaction is reversible and encompasses a positively charged surface region located in the vicinity of the heme IV. Overall, the study elucidates the formation of an electron transfer complex between PpcA and a readily outer-membrane permeable iron chelated form. The structural and functional relationships obtained explain how a single cytochrome is designed to effectively interact with a wide range of *G. sulfurreducens* electron acceptors, a feature that can be explored for optimal bioelectrochemical applications.

## Introduction

*Geobacter* species are highly abundant in natural subsurface environments where they can exchange electrons with insoluble acceptors, such as iron and manganese minerals (Reguera et al., 2005) or even with electrode surfaces (Holmes et al., 2004). The reduction of non-membrane permeable extracellular acceptors requires electrons to be channeled across the outer membrane. To date, the iron respiration electron transfer pathways in the bacterium *Geobacter sulfurreducens* are the best studied (Morgado et al., 2012b; Levar et al., 2014; Liu et al., 2014; Zacharoff et al., 2016). However, and despite the identification over the last decade of several key multiheme *c*-type cytochromes for reduction of iron-containing acceptors, the precise molecular mechanisms remain unknown. The identified group of cytochromes include the quinol oxidases ImcH and CbcL associated to the inner membrane (Levar et al., 2014; Zacharoff et al., 2016), a family of five triheme periplasmic cytochromes (PpcA-E) designated PpcA-family (Pokkuluri et al., 2004a; Morgado et al., 2010a) and porin-cytochrome *trans*-outer membrane complexes (Liu et al., 2015).

The conceptual model for the extracellular electron transfer in *G. sulfurreducens* considers that different proteins are involved in oxidation of the inner membrane quinols depending upon the redox potential of the final electron acceptor: the CbcL-dependent pathway operates at or below redox potentials of -100 mV (relative to the Normal Hydrogen Electrode, NHE), whereas the ImcH-dependent pathway operates above this redox potential (Levar et al., 2014; Zacharoff et al., 2016). In each case, electrons are supplied from the quinol oxidases to the PpcA-family cytochromes. The electrons are then transferred from periplasmatic to outer membrane associated cytochromes, through porin-cytochrome *trans*-outer membrane complexes toward extracellular acceptors. Whatever is the active respiratory pathway, the periplasmic cytochromes are always present and particularly the cytochrome PpcA, which is the most abundant cytochrome in *G. sulfurreducens* (Ding et al., 2008; Morgado et al., 2010a). This cytochrome has 71 residues and contains three covalently bound low-spin heme groups with bis-histidinyl axial coordination, which are diamagnetic (*S* = 0) and paramagnetic (*S* = 1/2) in the reduced and oxidized forms, respectively (Morgado et al., 2010b; Dantas et al., 2011). It is the most likely reservoir of electrons destined for outer surface (Lloyd et al., 2003; Ding et al., 2006) and it may also contribute to the periplasmic electron storing if external acceptors are temporary unavailable (Esteve-Núñez et al., 2008; Fernandes et al., 2017). The cytochrome PpcA from *G. sulfurreducens* is extremely well characterized both structurally and functionally (Pokkuluri et al., 2004a, 2010; Morgado et al., 2010a, 2012c, 2017). Interacting interface regions between this cytochrome and electron acceptors were recently characterized, using a set of complementary biophysical techniques including stopped-flow kinetics, molecular docking, UV-visible and NMR measurements. These studies allowed to identify the molecular complexes established between cytochrome PpcA and anthraquinone-2,6-disulfonate (AQDS), anthrahydroquinone-2,6,-disulfonate (AH_2_QDS) and Fe(III)-citrate (Dantas et al., 2014a, 2015; Ferreira et al., 2017). The results obtained also suggested that the cytochrome PpcA may function as terminal reductase for AQDS and Fe(III)-citrate fractional populations that are able to transverse the outer membrane and reach the periplasm. However, genetic and proteomic studies showed that the reduction of the humic substance analog (AQDS) and Fe(III)-citrate by *G. sulfurreducens* and *Shewanella oneidensis* cells is essentially carried out by the outer membrane cytochromes (Gescher et al., 2008; Voordeckers et al., 2010; Bucking et al., 2012; Liu et al., 2015), suggesting that the direct reduction of these acceptors by PpcA is not significant.

Ferric nitrilotriacetate (Fe-NTA) can also be utilized by *G. sulfurreducens* and *S. oneidensis* as electron acceptor (Coppi et al., 2004). Fe-NTA is found in several natural environments, particularly in sewage or contaminated waters (Edwards et al., 2012). Chelators, such as NTA, have an important role in the solubilization of Fe(III) and concomitant enhancement of its bioavailability (Lovley and Woodward, 1996). The stimulation of Fe(III) reduction may be used in several bioremediation applications, including the contaminant degradation of aromatic hydrocarbons in sediments from petroleum-contaminated aquifers (Lovley et al., 1994). This iron chelated form is smaller and more outer-membrane permeable compared to Fe(III)-citrate (Bucking et al., 2012). Therefore, it is expected that periplasmic reduction of Fe-NTA would be considerably higher compared to Fe(III)-citrate. This was in fact confirmed by Gescher et al. (2008) using a synthetic biology approach involving the reconstruction of the putative electron transport chain of the bacterium *S. oneidensis* in *Escherichia coli*. In addition, it was also shown that *S. oneidensis* outer membrane cytochromes contribute to the global reduction of Fe-NTA (Wang et al., 2008). Thus, given the cellular architecture similarity between *S. oneidensis* and *G. sulfurreducens*, it is conceivable that, in addition to periplasmic cytochromes, *G. sulfurreducens* outer membrane cytochromes also contribute to the reduction of Fe-NTA.

To provide evidence that PpcA can also function as terminal reductase and further contribute to the understanding of soluble iron respiratory pathways in *G. sulfurreducens*, we used UV-visible and NMR spectroscopic techniques to investigate the biomolecular interactions between PpcA and Fe-NTA and to map the interface interacting regions in the redox complex. The results obtained allowed us to identify a specific region of interaction between the two molecules. Overall, the information obtained provides important foundations to the understanding of the iron respiratory pathways in *G. sulfurreducens* and can be explored for optimal bioelectrochemical applications.

## Materials and Methods

### Expression and Purification of Cytochrome PpcA From *G. sulfurreducens*

Natural abundance and ^15^N-isotopic labeled samples of cytochrome PpcA were expressed and purified as previously described (Londer et al., 2002; Fernandes et al., 2008).

### UV-Visible Experiments

All the solutions were prepared in anaerobic conditions inside a glove box chamber, as previously described (Dantas et al., 2014a). Likewise, all the UV-visible absorption spectra were recorded in anaerobic conditions in the range 350–600 nm at 25°C (Dantas et al., 2014a).

Protein samples (3 μM) were prepared in phosphate buffer pH 7.0 with NaCl (100 mM final ionic strength). A concentrated Fe-NTA solution (3 mM) was prepared in the same buffer, accordingly to the procedure described by Bunescu et al. (2008). Reduction of PpcA was obtained by adding a stoichiometric amount of sodium dithionite.

### NMR Studies

For NMR studies, samples of PpcA were prepared in the buffer described above in 92%H_2_O/8% ^2^H_2_O (^15^N-labeled sample) or pure ^2^H_2_O (unlabelled sample). All the NMR experiments were performed in a Bruker Avance 600 MHz spectrometer at 25°C. The water signal was used to calibrate the ^1^H chemical shifts and the ^15^N shifts were calibrated through indirect referencing (Wishart et al., 1995). Data were processed using TOPSPIN software (Bruker Biospin, Karlsruhe, Germany) and analyzed with the program Sparky (TD Goddard and DG Kneller, Sparky 3, University of California, San Francisco, United States). The following set of experiments was acquired: (i) 2D ^1^H,^15^N-HSQC for the ^15^N labeled sample in 92%H_2_O/8% ^2^H_2_O, and (ii) 2D ^1^H,^13^C-HSQC; 2D ^1^H,^1^H-NOESY with 80 ms mixing-time and 1D ^1^H-NMR spectra in pure ^2^H_2_O. The backbone NH and heme methyl signals of PpcA were previously assigned (Morgado et al., 2010b; Dantas et al., 2011, 2014a) and were used to monitor the effect of the addition of Fe-NTA. The effect of Fe-NTA addition on the heme signals was monitored by the analysis of a series of ^1^H-NMR spectra. This effect was also studied via 2D ^1^H,^13^C-HMQC and 2D ^1^H,^1^H-NOESY obtained in the absence and presence of PpcA (1:3 PpcA:Fe-NTA ratio). The effect of the addition of Fe-NTA in the PpcA cytochrome backbone NH signals was monitored by recording 2D ^1^H,^15^N-HSQC NMR spectra in the absence and presence of PpcA (1:3 PpcA:Fe-NTA ratio). The pH of the samples was measured before and after each NMR spectra series. To confirm protein integrity 1D ^1^H-NMR spectra were acquired before and after each 2D spectrum.

Finally, to investigate the binding reversibility, 1D ^1^H-NMR spectra were acquired for free PpcA, 1:3 PpcA:Fe-NTA ratio and after removal of Fe-NTA by ultrafiltration using Amicon Ultra devices with a 3 kDa molecular weight cutoff.

## Results and Discussion

### Probing the Reduction of Fe-NTA With Cytochrome PpcA by UV-Visible Spectroscopy

**FIGURE 1:**
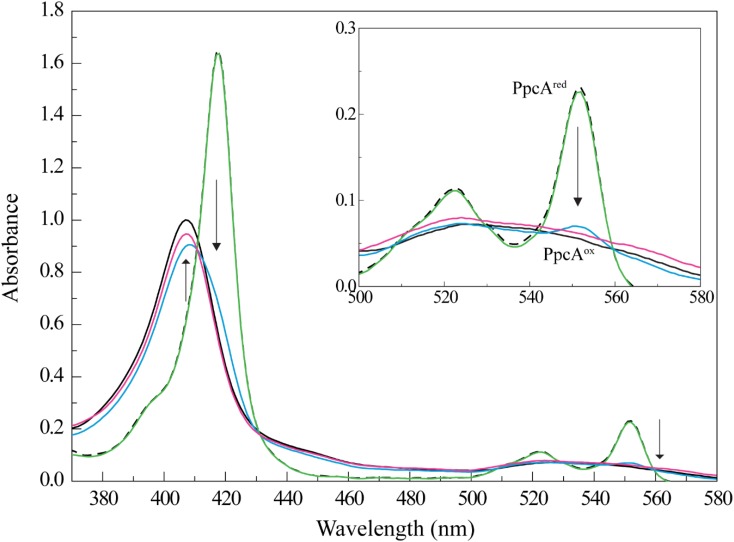
PpcA oxidation by Fe-NTA monitored by UV-visible spectroscopy at 25°C and pH 7. The UV-visible spectra of PpcA in the oxidized (PpcA^ox^) and reduced (PpcA^red^) forms are indicated by the continuous and dashed black lines, respectively. The concentration of PpcA used in the experiments was 3 μM. Spectra obtained for PpcA:Fe-NTA ratios are indicated by colored lines: 1:1 (green), 1:3 (blue), and 1:10 (pink). The arrow points to increasing concentrations of Fe-NTA. For clarity, an enlarged scale of the spectra between 500 and 580 nm is showed in the inset.

The different UV-visible absorption spectral features of cytochrome PpcA in the reduced and oxidized forms were explored to study the reduction of Fe-NTA (Figure 1). In fact, the UV-visible spectrum of PpcA in the oxidized state showed a peak at 406 nm (Soret band) and a very broad peak in the range 510–570 nm. On the other hand, three distinct bands are clearly visible in the reduced form: 417 nm (Soret band), 522 nm (β band), and 552 nm (α band). Thus, we monitored the effect of the addition of a Fe-NTA solution to a reduced PpcA sample. With the concomitant addition of Fe-NTA the characteristic bands of the reduced cytochrome disappear and are replaced by those of the oxidized form, confirming that PpcA is able to reduce Fe-NTA (Figure 1).

Compared to other iron chelated forms, the equilibrium speciation in solution of Fe-NTA at pH 7.0 is less complex and essentially one main iron form is present: [FeOH-NTA]^-^ (92%) *versus* [Fe(OH)_2_-NTA]^2-^ (8%) (Wang et al., 2008). The higher reduction potential of the dominant iron form, +0.372 mV, (Thamdrup, 2000) compared to those of PpcA heme groups: -147, -104, and -111 mV for hemes I, III, and IV, respectively (Santos et al., 2015) explains the favorable reduction of FeOH-NTA^-^ by the cytochrome. The negative reduction potential of PpcA heme groups indicates that the electron acceptor can be reduced by any of them. To elucidate if one or more heme groups are directly involved in the reduction of Fe-NTA, we then studied the molecular interactions between the two molecules by NMR.

### NMR Interaction Studies Between PpcA and Fe-NTA Probed by NMR

Nuclear Magnetic Resonance chemical shift perturbation experiments are extremely powerful to identify protein–protein or protein-ligand complexes by providing wealthy structural information about the nature of the complex interacting interfaces. Theoretically, NMR chemical shift perturbation experiments can be monitored by any type of NMR spectrum. However, the 2D ^1^H,^15^N-HSQC NMR spectra are the most popular ones. In fact, these experiments correlate the chemical shifts of the nitrogen nuclei, in a ^15^N-labeled protein, with their directly attached protons. Thus, as every amino acid contains a peptide bond NH group, except proline residues, 2D ^1^H,^15^N-HSQC NMR experiments obtained for the cytochrome sample in the absence and presence of its putative partner(s) will provide a good fingerprint of the binding interface. Indeed, upon complex formation the chemical environment of the interacting residues will be affected and, as a consequence, their NMR signals. In the case of a protein-ligand complex, these changes can be reported by chemical shift variations of the affected residues and/or by the line width broadenings. The latter is observed in case of paramagnetic ligands whose unpaired electrons decrease the relaxation rates and increase the line widths of interacting groups in the protein (for a review see Bertini and Luchinat, 1986).

However, in the case of cytochromes, the study of molecular interactions is further complex due to the substantial differences in the NMR spectra of the diamagnetic and paramagnetic forms. Indeed, in the oxidized state, the paramagnetic effect of the unpaired electron(s) at the heme iron spreads the heme substituent NMR signals and neighboring residues over larger spectral widths, compared to the reduced and diamagnetic state. In fact, in the particular case of cytochrome PpcA from *G. sulfurreducens*, the protein signals are spread over a spectral window between -5 to 21 ppm in the oxidized state, whereas in the reduced state they cover a substantial smaller spectral window (-2 to 11 ppm) (Salgueiro and Dantas, 2017). Therefore, the study of the interacting regions between PpcA and its putative redox partners cannot be accomplished by the addition of increasing amounts of the electron acceptor in the oxidized state to PpcA in the reduced form. If this was the case, the NMR spectral changes due to the formation of the redox complex will be masked by the more significant redox-linked signal spectral changes. For this reason, the study of the interacting regions in redox partners is carried out by keeping them in the same oxidation state (Prudencio and Ubbink, 2004; Yahata et al., 2006; Meschi et al., 2011; Sakamoto et al., 2011).

The solution structures of PpcA have been determined in the fully oxidized and fully reduced forms (Morgado et al., 2012c, 2017). The structures are highly conserved suggesting that the interacting regions with putative redox partners can be mapped in experiments carried out with the protein in the reduced or oxidized state. This was indeed confirmed by NMR chemical shift perturbation studies between PpcA and the humic substance analog, which revealed that this molecule interacts in the same region with the cytochrome (Dantas et al., 2014a, 2015).

From the above, and given the smaller NMR signal overlap in the oxidized state, the molecular interactions between PpcA and the electron acceptor Fe-NTA were investigated in this oxidation state. 2D ^1^H,^15^N-HSQC experiments were used to probe the chemical shift perturbations at the PpcA polypeptide backbone NH signals. These signals have been previously assigned (Dantas et al., 2014a). All signals were assigned except those of Ala^1^ and Asp^2^ due to fast exchange with the solvent; Gly^36^ and Gly^42^ due to signal broadening caused by the nearby hemes paramagnetic irons and Cys^51^ due to its rapid solvent exchange.

**FIGURE 2:**
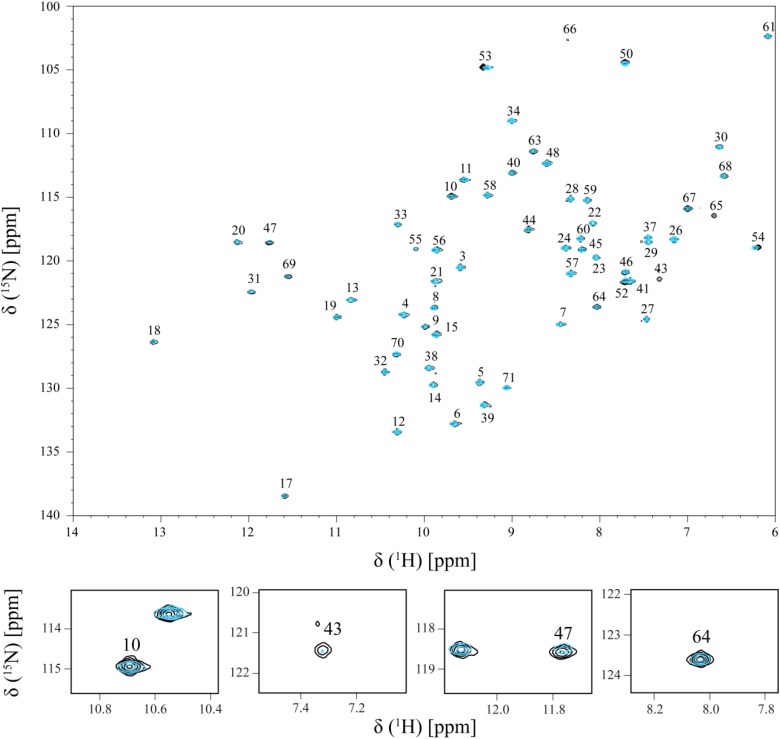
Overlay of the 2D ^1^H,^15^N-HSQC NMR spectra of ^15^N-enriched PpcA sample acquired in the absence (black) and in the presence (cyan) of Fe-NTA. The spectrum in presence of Fe-NTA was obtained for a PpcA:Fe-NTA ratio of 1:3. The assignment of the NH signals is indicated. For clarity, expansions of the most affected signals are shown at the bottom of the figure.

**FIGURE 3:**
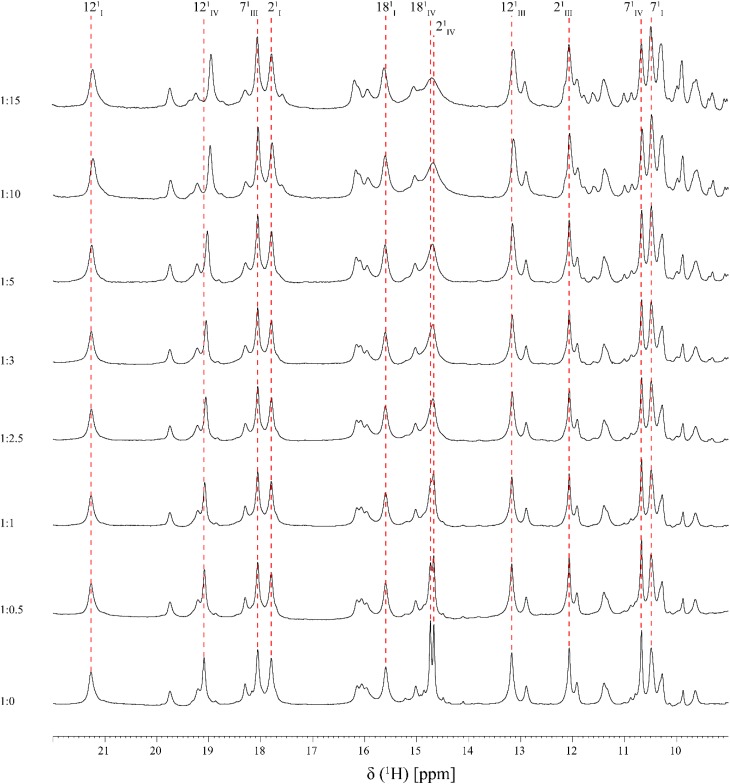
Expansions of the low-field region of 1D ^1^H-NMR spectra obtained for PpcA in the presence of increasing amounts of Fe-NTA (pH 7 and T 25°C). The heme methyls labeled accordingly to the IUPAC nomenclature for tetrapyrroles (Moss, 1988) are indicated, with exception of the 18^1^CH_3_^III^ signal which appears at a chemical shift of 0.76 ppm. For historical reasons and to be consistent with the literature the three heme groups of PpcA are numbered I, III, and IV, due to their structural superimposable arrangement with those of the homologous tetraheme cytochromes *c*_3_ (Pessanha et al., 2004).

**FIGURE 4:**
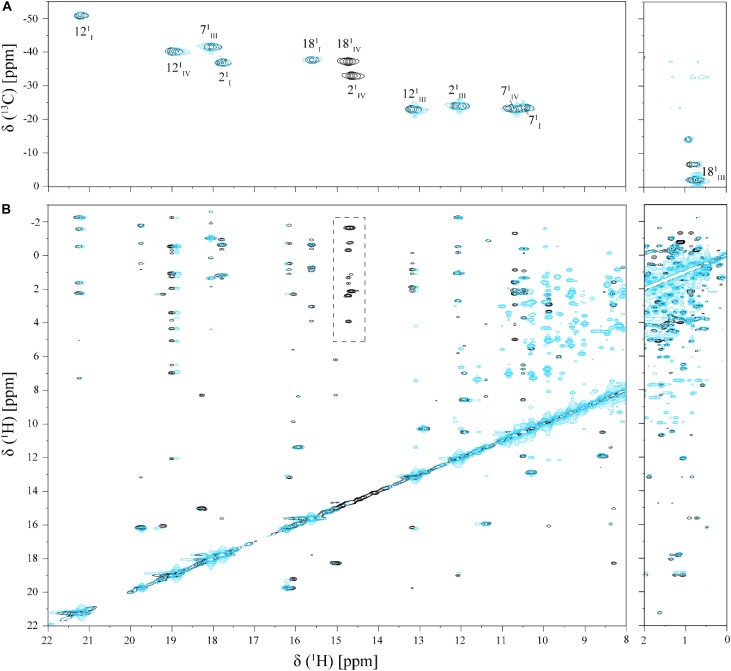
Selected regions of 2D ^1^H,^13^C-HMQC **(A)** and 2D ^1^H,^1^H-NOESY **(B)** NMR spectra of cytochrome PpcA in the absence (black) and presence (cyan) of Fe-NTA (pH 7 and T 25°C). The dashed rectangle shows the NOE connectivities of heme methyls 2^1^CH_3_^IV^ and 18^1^CH_3_^IV^, which presents significant broadening in the presence of Fe-NTA.

The effect of the addition of Fe-NTA on PpcA NH signals is illustrated in Figure 2. The comparison of the 2D ^1^H,^15^N-HSQC NMR spectra acquired in the absence and presence of Fe-NTA indicates that the free and bound structures of PpcA are similar on side chains position since the spectral dispersion of the NMR signals is maintained (Figure 2; see also Figures 3, 4). However, important signal line width broadening as well as chemical shift variation though at a lesser extent, was observed upon addition of Fe-NTA. The increase in the line width broadening, as a function of Fe-NTA concentration, is explained by the paramagnetic high spin (*S* = 5/2) character of the ferric iron (Kawabata et al., 1986) and concomitant decrease in the relaxation rates of the neighboring nuclei.

As shown by the UV-visible experiments, Fe-NTA can be reduced by the cytochrome PpcA. Therefore, to assure an effective electron transfer within the redox complex, at least one heme group must be in the vicinity of the acceptor. However, due to the similar reduction potential values of PpcA heme groups, the electron acceptor can be putatively reduced by any heme. As a consequence of the small size of cytochrome PpcA, which displays a ratio of 24 residues per heme group, it is expected that any perturbation caused by binding of the Fe-NTA would affect the heme substituent NMR signals. The heme methyl group signals are found in less crowded regions of the NMR spectra and therefore are the best heme substituents to monitor this effect (see Figure 3). All the heme methyl signals of PpcA were previously assigned (Morgado et al., 2010b) and both 1D ^1^H and 2D ^1^H-NOESY NMR spectra were used to further evaluate the molecular interactions between Fe-NTA and PpcA. Figure 3, indicates the region of 1D ^1^H NMR spectra that contains the heme methyl signals, except 18^1^CH_3_^III^, which appears at 0.76 ppm. Since this signal cannot be followed by 1D ^1^H NMR titrations, the evaluation of the addition of Fe-NTA on the full set of heme methyl signals was monitored by 2D ^1^H,^13^C-HMQC NMR spectra recorded in the absence and presence of the electron acceptor (Figure 4A). The analysis of the referred spectra confirms that the protein conformation is conserved in the presence of Fe-NTA since the chemical shift dispersion of the signals is maintained. In addition, there is a significant line width broadening of the heme IV methyl signals. The heme methyls 2^1^CH_3_^IV^ and 18^1^CH_3_^IV^ are clearly the most affected ones compared to 7^1^CH_3_^IV^ and 12^1^CH_3_^IV^ signals. These observations were further confirmed by an analysis of the heme methyl NOE connectivities (Figure 4B).

**FIGURE 5:**
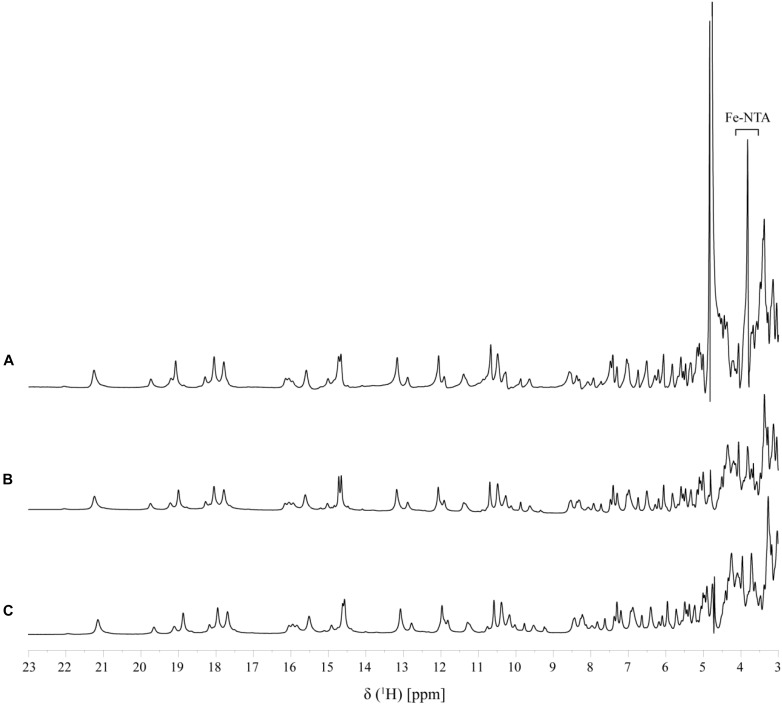
Reversibility of Fe-NTA binding to PpcA monitored by 1D ^1^H-NMR (pH 7 and T 25°C). A fingerprint region of the PpcA 1D ^1^H-NMR spectrum is shown: **(A)** PpcA in the presence of Fe-NTA (PpcA:Fe-NTA ratio 1:0.5); **(B)** PpcA prior to the addition of Fe-NTA, and **(C)** PpcA after removal of Fe-NTA.

In order to assure directionality in the reduction of the electron acceptor it is important that the interaction is reversible. This was evaluated by the comparison of 1D-^1^H NMR spectra of PpcA recorded before and after the removal of the electron acceptor by ultrafiltration, which confirms that the interaction is fully reversible (Figure 5).

### Structural Rationale for the Interface Interacting Region Between PpcA and Fe-NTA

**FIGURE 6:**
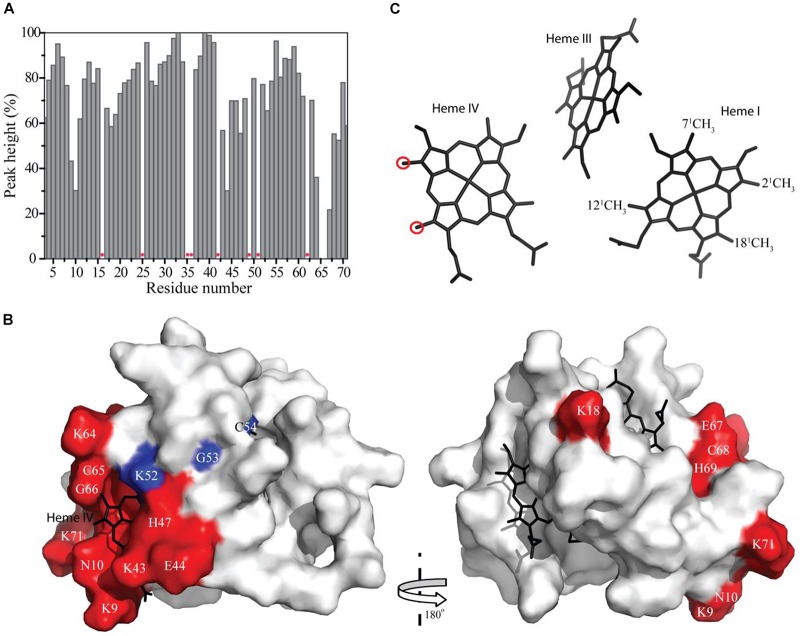
Line width broadening and chemical shift peturbations on PpcA from *Geobacter sulfurreducens* NMR signals. **(A)** Variation of the peak height of the PpcA signals (%) measured from the 2D ^1^H,^15^N-HSQC NMR spectra indicated in Figure 2. Red asterisks indicate proline or non-assigned residues. **(B)** Surface mapping of the most affected residues. The red and blue residues correspond to those that showed a decrease in the peak data height above 40% and a significant deviation in their chemical shift, respectively. The heme groups are represented in black. The molecular surface was generated in Pymol (Schrodinger, 2015) using the structure of PpcA [PDB code: 2LDO (Morgado et al., 2012c)]. The left and right panels are related by a 180° rotation. **(C)** Heme core of PpcA [PDB code: 2LDO (Morgado et al., 2012c)]. Red circles indicate the most affected heme methyl signals (see also Figure 3).

The residues whose NH backbone signals showed a decrease in the peak data height above 40% are Lys^9^, Asn^10^, Lys^18^, Lys^43^, Glu^44^, His^47^, Lys^64^, Cys^65^, Gly^66^, Glu^67^, Cys^68^, His^69^, and Lys^71^ (Figure 6A). The most affected residues included the PpcA heme IV binding motif (Cys^65^-Gly^66^-Glu^67^-Cys^68^-His^69^) and, except for Lys^18^, all the others are located near this heme (Figure 6B). A small set of sequential residues (Lys^52^, Gly^53^, and Cys^54^) experienced a considerably smaller line width broadening (Figure 6A) but chemical shift perturbation was observed in their NH signals (see also Figure 2). These three residues were also mapped on the structure of PpcA and their proximity to heme IV increases from Lys^52^ to Cys^54^ (see blue colored residues in Figure 6B). Therefore, the observed effect probably reflects the propagation of perturbations from the binding surface closer to heme IV (see red colored residues in Figure 6B) rather than direct binding.

The effect of the addition of Fe-NTA on PpcA monitored by the heme methyl signals also confirms the interaction between the two molecules in the vicinity of heme IV. In fact, the most solvent exposed heme methyls (2^1^CH_3_^IV^ and 18^1^CH_3_^IV^) are clearly the most affected ones (see Figure 6C).

## Conclusion and Implications

In the present work, we showed that Fe-NTA can be directly reduced by the triheme cytochrome PpcA from *G. sulfurreducens*. The results obtained are in accordance with the reduction potential values of both molecules at pH 7. The reduction potential values of the three heme groups of PpcA cover the functional range -147 to -104 mV (Santos et al., 2015), whereas that of Fe-NTA is considerably higher (+0.372 mV), favoring the thermodynamic reduction of the latter. Since any of the PpcA heme groups can hypothetically reduce the electron acceptor, we also probed the molecular interaction between the cytochrome and the Fe-NTA by NMR. The results showed that the cytochrome interaction region comprises a high number of positively charged lysine residues located in the vicinity of the most exposed edge of heme IV. However, the other two hemes are also considerably exposed to the solvent (Pokkuluri et al., 2004b; Morgado et al., 2012c), which anticipates that the interaction between the Fe-NTA molecule and PpcA heme IV is directed by other factors. Considering the negative charge of the most abundant form of the Fe-NTA in solution at pH 7.0 (FeOH-NTA^-^), electrostatic interactions driving by the positively charged lysine residues in the vicinity of heme IV are expected to favor the formation of the PpcA:FeOH-NTA^-^ redox complex.

**FIGURE 7:**
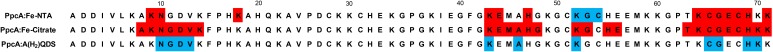
Comparison of the most affected residues in the redox complexes established between PpcA from *G. sulfurreducens* and the elctron acceptors Fe-NTA (this work), Fe-citrate (Ferreira et al., 2017) and AQ(H_2_)DS (Dantas et al., 2014a, 2015). The residues highlighted in red and blue correspond to those that showed a decrease in the peak data height above 40% or a significant deviation in their chemical shift, respectively.

Interestingly, previous studies have also shown that the heme IV region in the cytochrome PpcA from *G. sulfurreducens* was also involved in the formation of redox complexes with other electrons acceptors, namely the humic substance analog (AQDS) and Fe-citrate (Dantas et al., 2014a, 2015; Ferreira et al., 2017). A comparison of the interacting polypeptide regions between PpcA and the three electron acceptors are summarized in Figure 7. Just as the dominant species of Fe-NTA at pH 7.0, the charge of these two electron acceptors is also negative. Therefore, the available data for the molecular interacting regions between PpcA and different negatively charged *G. sulfurreducens* terminal electron acceptors unveils the structural and functional features underlying the effective formation of the redox complexes and concomitant reduction of the different electron acceptors. Indeed, in order to efficiently reduce the electron acceptor, the heme directly involved in this reduction must have (i) a lower reduction potential value compared to those of the electron acceptors and (ii) a positively charged surface in its vicinity to electrostatically assist in the redox complex formation. Thus, this two observations can putatively be used as starting models to assist the optimization of the molecular interaction between cytochromes that could interact with negatively charged electron acceptors by enhancing their reactivity and to improve biotechnological applications with engineered *Geobacter* strains with optimal desirable properties for the bioremediation of environmental organic and metal contaminants (Fredrickson and Gorby, 1996). In addition, the ability of cytochrome PpcA to reduce toxic and radioactive compounds (Renshaw et al., 2005) coupled to its robustness, has been shown by its high stability (Tm > 85°C) (Pokkuluri et al., 2010) and maintenance of its mechanistic and functional features over a wide range of pH and ionic strength values (Morgado et al., 2012a; Dantas et al., 2014b), pave the way to develop rational PpcA-based bioelectrochemical strategies to the bioremediation of toxic/radioactive metals.

## Author Contributions

MF carried out the experiments, interpreted the data, and wrote the manuscript. CS designed the study, discussed the data, and wrote the manuscript.

## Conflict of Interest Statement

The authors declare that the research was conducted in the absence of any commercial or financial relationships that could be construed as a potential conflict of interest.
